# Intersection of Tyrosine Kinase Inhibition and Sepsis‐Induced Cardiomyopathy

**DOI:** 10.1155/crip/7134352

**Published:** 2025-12-29

**Authors:** Hiroshi Abe, Kiyoshi Takasu, Kei Yamamoto, Tomita Shigeki, Tetsuro Miyazaki, Takashi Tokano, Tohru Minamino

**Affiliations:** ^1^ Department of Cardiology, Juntendo Daigaku Igakubu Fuzoku Urayasu Byoin, Urayasu, Chiba Prefecture, Japan; ^2^ Department of Clinical Pathophysiology, Tokyo Shika Daigaku, Chiyoda, Tokyo, Japan; ^3^ Department of Pathology, Juntendo Daigaku Igakubu Fuzoku Urayasu Byoin, Urayasu, Chiba Prefecture, Japan; ^4^ Department of Cardiovascular Biology and Medicine, Juntendo Daigaku Igakubu Daigakuin Igaku Kenkyuka, Bunkyo, Tokyo, Japan

**Keywords:** old myocardial infarction, sepsis-induced cardiomyopathy, tyrosine kinase inhibitor

## Abstract

This case details a 78‐year‐old male with a history of old myocardial infarction and chronic myeloid leukemia treated with tyrosine kinase inhibitors (TKIs). The patient presented with exertional dyspnea and was hospitalized for congestive heart failure. Following the development of a urinary tract infection, the patient exhibited signs of sepsis‐induced cardiomyopathy (SIC), including myocardial injury and rapid hemodynamic deterioration, leading to death. Autopsy findings indicated SIC, characterized by neutrophil‐dominant inflammatory cell infiltration and endothelial damage, which might have been exacerbated by the patient′s preexisting conditions of old myocardial infarction and TKI therapy. This case underscores the multifactorial nature of SIC, suggesting that preexisting old myocardial infarction and TKI therapies can significantly impact the disease′s pathogenesis and progression. It highlights the need for comprehensive management strategies in patients with complex medical histories and the importance of further research to elucidate the underlying mechanisms of SIC.

## 1. Introduction

Sepsis‐induced cardiomyopathy (SIC) is a condition characterized by myocardial cell impairment triggered by sepsis [1]. Despite implications for patient outcomes, no formal definitions exist for septic cardiomyopathy. Most review articles and expert opinions agree on a few fundamental features of this unique form of cardiac dysfunction, which are outlined as follows: The condition is characterized by its acute and reversible nature, typically resolving within 7–10 days; it manifests as global, biventricular dysfunction with a notable reduction in contractility; left ventricular dilatation is a distinctive aspect of this cardiac dysfunction; there is a diminished response to both fluid resuscitation and catecholamines; notably, the etiology does not involve acute coronary syndrome [2]. Many aspects of the mechanisms and pathophysiology of SIC remain unclear. Conversely, tyrosine kinase inhibitors (TKIs) are medications that can potentially cause vascular impairment [3]. In this case, we report the occurrence of SIC in a patient undergoing TKI therapy, revealing distinctive pathological findings upon autopsy.

## 2. Case Description

The patient, a 78‐year‐old male, presented to the outpatient clinic with a chief complaint of exertional dyspnea persisting for 1 week. His medical history included hypertension, dyslipidemia, prior pulmonary tuberculosis, bronchial asthma, and past rectal cancer surgery, but he has not received chemoradiotherapy. Additionally, 8 years ago, he underwent percutaneous coronary intervention (PCI) with drug‐eluting stent placement in the left anterior descending artery due to acute anterior myocardial infarction. Then, 6 months prior, catheter intervention using a drug‐coated balloon and drug‐eluting stent was performed for in‐stent restenosis of the left anterior descending artery. He also underwent catheter intervention for peripheral artery disease with a drug‐coated balloon in both superficial femoral arteries 3 months ago. For chronic myeloid leukemia, he initially underwent dasatinib hydrate treatment for 1 year. Still, the peripheral blood polymerase chain reaction (International Scale) was 0.2526, showing a partial response instead of a complete cytogenetic response. Consequently, the treatment was switched to ponatinib, and he has been on ponatinib therapy for the past 2 years. Other medications included clopidogrel 75 mg, rivaroxaban 15 mg, atorvastatin 10 mg, bisoprolol 2.5 mg, lansoprazole 15 mg, and amlodipine 10 mg. He had a history of smoking (20 cigarettes per day) until his acute myocardial infarction 8 years ago. Still, he did not have an alcohol habit, and there was no family history of cardiovascular diseases.

Upon presentation, his height was 157 cm, weight was 55 kg, and body mass index was 22.3 kg/m^2^. Vital signs showed a pulse rate of 58 bpm, blood pressure of 170/68 mmHg, and a temperature of 36.5°C. Bilateral lung auscultation revealed intermittent crackles, and a gallop rhythm was audible on cardiac auscultation. Mild pitting edema was noted. An electrocardiogram displayed sinus rhythm with left‐axis deviation and abnormal *Q* waves in *V*_2_ and *V*_3_ alongside the right bundle branch block. Chest x‐ray revealed cardiomegaly, enhanced pulmonary vascular markings, interstitial edema, and Kerley C lines. Echocardiography indicated an ejection fraction of 58% with decreased wall motion in the anterior–septal region. Moderate mitral regurgitation and mild tricuspid regurgitation with a TR Vmax of 3.5 m/s were observed, indicative of pulmonary hypertension. Laboratory findings showed elevated BNP (412.6 pg/mL) and troponin T (0.068 ng/mL), along with hemoglobin at 11.3 g/dL, albumin at 4.1 g/dL, creatinine at 0.91 mg/dL, AST at 23 IU/L, ALT at 26 IU/L, eGFR at 62 mL/min, CK 91 IU/mL, and CRP 2.7 mg/dL.

The patient was admitted for congestive heart failure. Initial management included oxygen therapy, nicorandil infusion at 0.1 mg/kg/h as a vasodilator, and furosemide 20 mg intravenous injection twice daily for diuresis. Ponatinib was continued. Although initially responding well, the patient developed a fever on the second day due to a urinary tract infection, prompting piperacillin–tazobactam initiation. *Klebsiella*, a gram‐negative bacillus, was detected in the urine but not in the blood cultures. Subsequently, acute kidney injury was observed, marked by an increase in creatinine to 1.91 mg/dL. AKI was defined using Kidney Disease Improving Global Outcomes (KDIGO) AKI criteria [4]. Prerenal acute kidney injury was suspected based on urinalysis findings. It was attributed to sepsis, resulting in a Sequential Organ Failure Assessment (SOFA) score of 5 points. Emphasizing these findings, a diagnosis of sepsis was confirmed based on the elevated SOFA score. Diuretics were discontinued, and fluid resuscitation was performed, leading to recovery from acute kidney injury by the fifth day (creatinine: 1.59 mg/dL) alongside the peak of inflammation (CRP: 22 mg/dL).

However, on the sixth day, there was a rise in creatine kinase (CK) 1046 IU/L, CKMB (117 ng/mL), and troponin T 2.030 ng/mL, indicating myocardial injury, accompanied by marked peripheral coldness and oliguria. No evident chest symptoms or significant ST changes were observed on the electrocardiogram. The patient was transferred to the intensive care unit due to sepsis for comprehensive management. Subsequent echocardiography showed no change in left ventricular contractility or new wall motion abnormalities. However, dilatation of the left ventricle chamber was noted. Despite the severity of the condition, the degree of tricuspid regurgitation and pulmonary hypertension remained unchanged. Peripheral venous congestion was stressed, but no respiratory variations were observed. The patient developed atrial fibrillation but remained adequately controlled with a heart rate below 110/min. Despite aggressive therapy with dobutamine and continuous renal replacement therapy, there was poor response, leading to further deterioration in respiratory status necessitating noninvasive positive pressure ventilation. Additionally, the patient required norepinephrine and vasopressin due to refractory hypotension. Unfortunately, the patient progressed to asystole on the eighth day and succumbed with no desire for resuscitation.

Given the elevation of myocardial enzyme and rapid deterioration in hemodynamics, an autopsy was proposed to the family to investigate the cause of myocardial impairment, which was consented to and subsequently conducted.

Regarding the pathological findings: In the left lung, metastasis from rectal cancer operated on 3 years ago revealed moderately differentiated tubular adenocarcinoma (tub2). Concerning the bone marrow, hyperplastic marrow was observed, but chronic myeloid leukemia was maintained under treatment with TKIs. The pulmonary artery exhibited fibrous thickening due to elastic fiber proliferation, with an average vessel diameter of 592 *μ*m (range: 96.1–1772 *μ*m) and changes noted in 26.6% of pulmonary arteries (Figure 1). Periodic acid methenamine (PAM) silver staining was utilized for renal examination, revealing endothelial enlargement and duplication of glomerular basement membranes with expansion of the subendothelial space (Figure 2). Partial inflammatory cell infiltration in the bladder, indicating signs of urinary tract infection, was considered a potential cause of sepsis.

Figure 1(a, b) Elastica van Gieson staining (EVG) revealing fibrous thickening by elastic fibers within the pulmonary artery.

**Figure:**
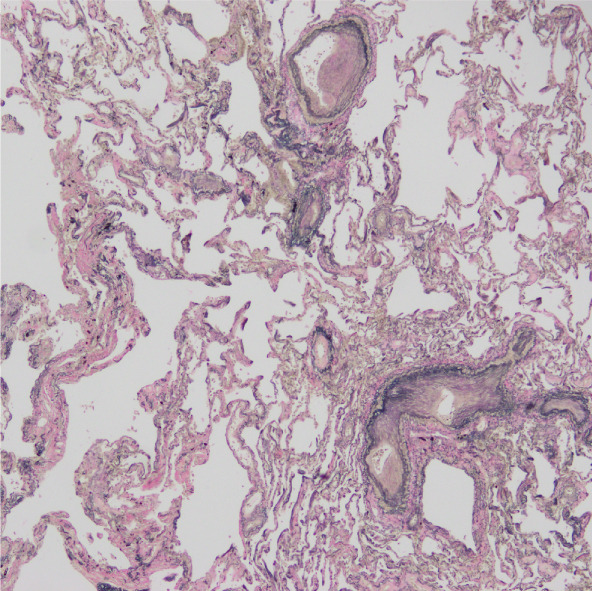
(a)

**Figure:**
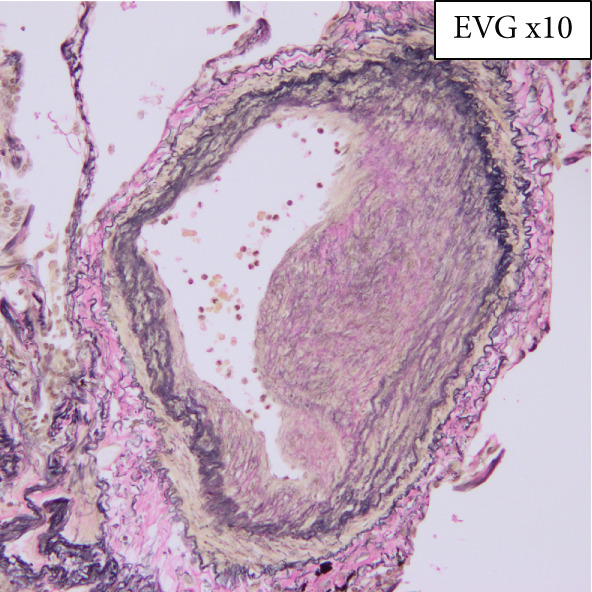
(b)

Figure 2(a) PAS highlighting segmental glomerular capillary wall thickening and remodeling (*yellow arrows*). (b) Periodic acid methenamine (PAM) silver staining demonstrating duplication of the glomerular basement membrane (*yellow arrows*).

**Figure:**
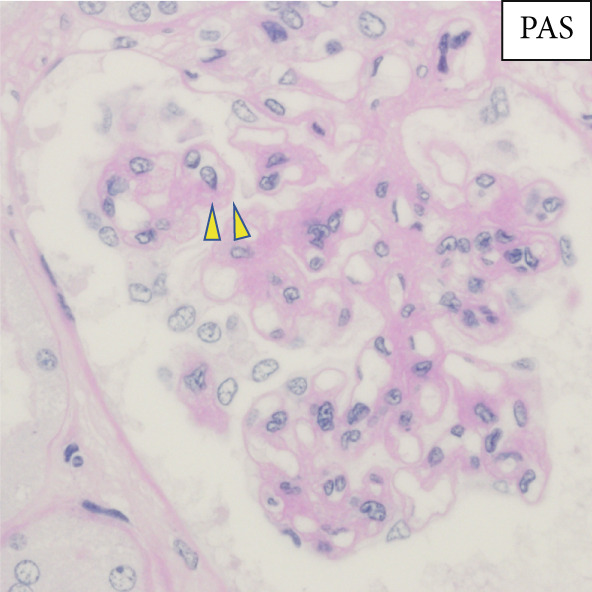
(a)

**Figure:**
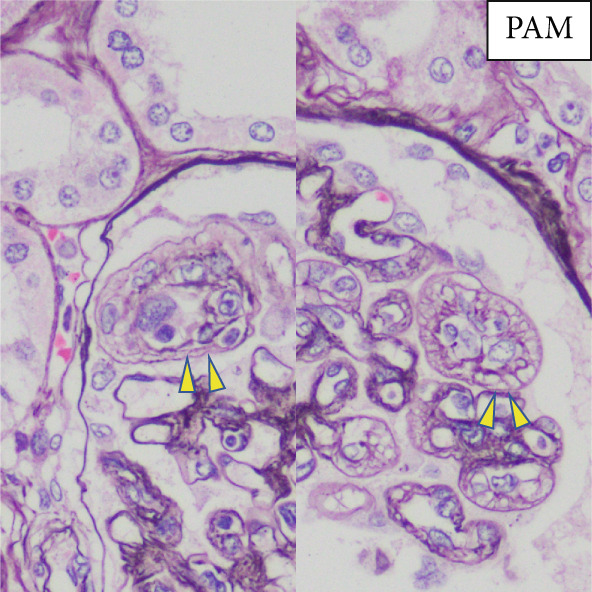
(b)

**Figure 4:**
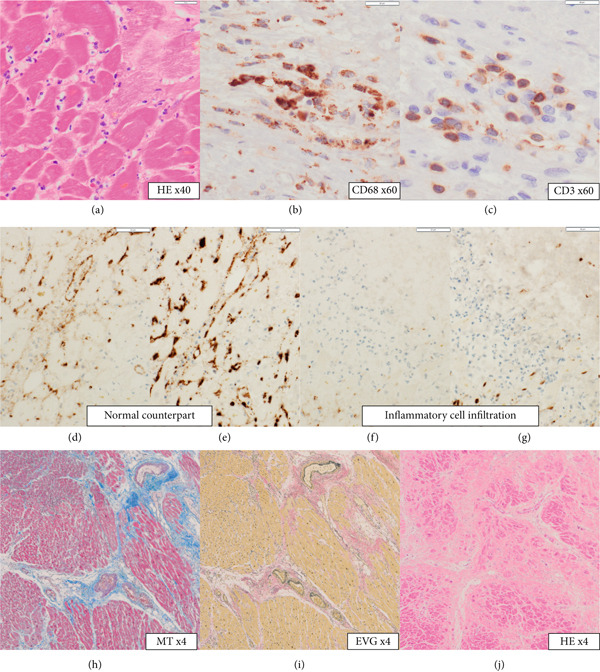
(a) H&E, × 40: overview of the inflammatory focus. (b) CD68, × 60: macrophage‐rich areas within the infiltrate. (c) CD3, × 60: admixed T‐cell infiltrates. (d, e) CD31, × 40 and CD34, × 40: preserved endothelial staining in vessels adjacent to less‐inflamed areas. (f, g) CD31, × 40 and CD34, × 40: attenuated endothelial staining in heavily inflamed regions, consistent with endothelial injury. (h) Masson′s trichrome, × 4: collagen deposition (blue) delineating fibrotic replacement in the infarct bed. (i) Elastica van Gieson (EVG), × 4: disruption of elastic fibers in affected vascular/structural elements, supporting postinjury remodeling. (j) H&E, × 4: highlighting maturing collagen bundles and reduced cellularity, consistent with progressing fibrosis.

The heart exhibited signs of old transmural infarction in the anterior septal region. However, no evidence of coronary artery occlusion or new thrombosis suggesting acute coronary syndrome was observed. Neutrophil‐dominant inflammatory cell infiltration was predominantly observed in the anterior septum, posterior, and lateral wall. There were no signs of colon cancer metastasis or leukemia cells. No evident bacterial components were observed with Gram staining. On high‐power H&E, small basophilic nuclear fragments with attenuated cytoplasm were identified at the inflammatory edge, suggestive of apoptotic bodies (Figure 3). Interstitial fibrosis, contraction band necroses, and interstitial edema were noted, along with partial CD68‐positive macrophages, consistent with previous reports of septic myocardiopathy [5], [6] In the inflammatory phenotype, CD68‐positive macrophages and CD3‐positive T cells were demonstrated. Endothelial markers (CD31/CD34) were preserved in less‐inflamed areas but became attenuated in heavily inflamed regions. Masson′s trichrome demonstrated collagen deposition, and EVG showed elastic fiber disruption, findings best interpreted as chronic fibrosis, most consistent with the old infarct substrate rather than hyperacute change (Figure 4).

Figure 3(a) Macroscopic view identifying infiltration of inflammatory cells (*highlighted in blue*). (b) H&E: left anterior descending artery and infarct area (*marked with asterisks*) (c) H&E, × 2: identification of inflammatory cell infiltration (*marked with asterisks*) (d) H&E, × 40: (e) H&E, × 60: in the right‐central field, two juxtaposed small basophilic nuclear fragments (5–7 *μ*m) with attenuated cytoplasm, consistent with cell shrinkage (pyknosis) and nuclear fragmentation (karyorrhexis) (*arrowheads*). Additional condensed, sharply marginated nuclear fragments are present in the right‐upper area. These features suggest apoptotic bodies at the edge of the inflammatory focus. (f) Gram staining does not reveal evident bacterial components.

**Figure:**
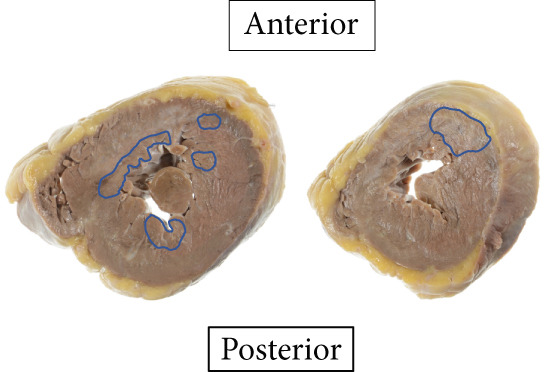
(a)

**Figure:**
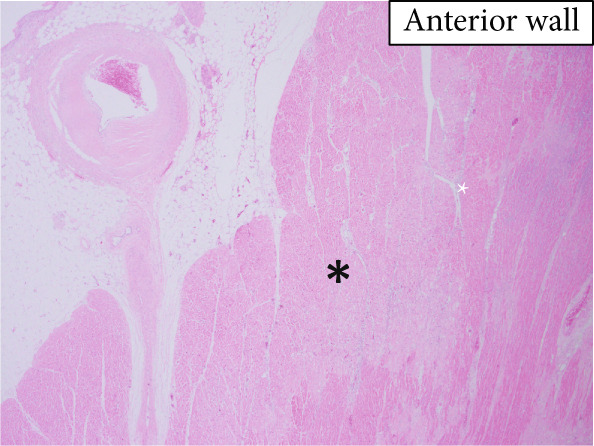
(b)

**Figure:**
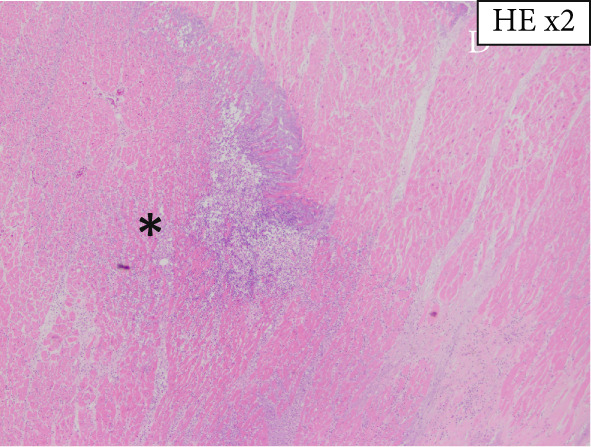
(c)

**Figure:**
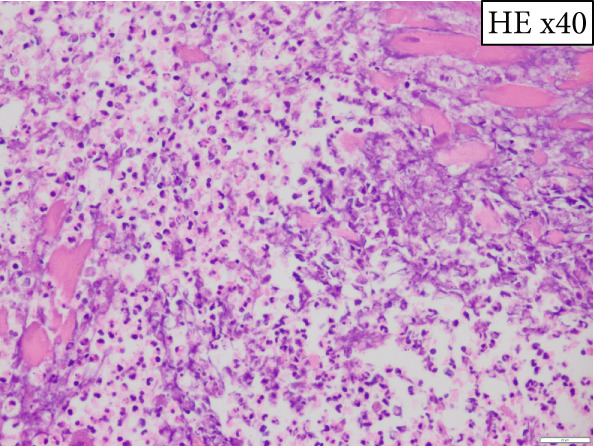
(d)

**Figure:**
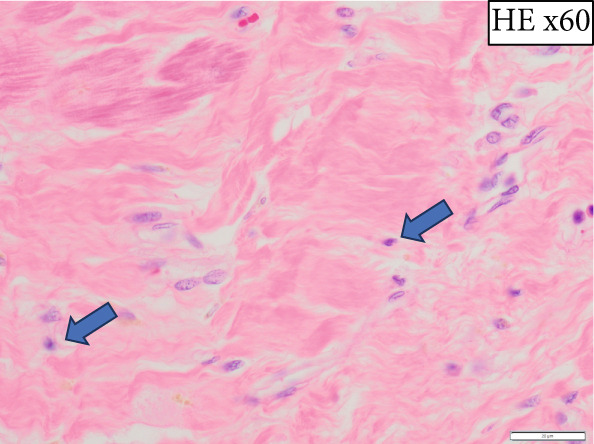
(e)

**Figure:**
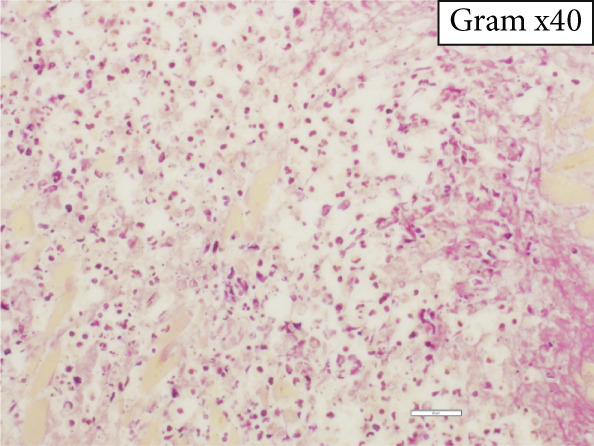
(f)

## 3. Discussion

The present case involves a patient admitted for heart failure who subsequently developed sepsis from a urinary tract infection. Then, 5 days after the onset of sepsis, an elevation in myocardial enzymes was observed, indicating myocardial injury. The patient exhibited biventricular heart failure. Response to fluid resuscitation and catecholamines was poor. The absence of pathological findings indicative of acute coronary syndrome, coupled with clinical course observations, aligned with the characteristics of septic cardiomyopathy. Therefore, based on the clinical course and pathological findings, the diagnosis of septic cardiomyopathy was established [2]. Regarding the present case, we discuss the pathophysiology of SIC.

This case exhibited numerous pathological features typical of septic cardiomyopathy, leading to death due to myocardial dysfunction and rapid hemodynamic deterioration. Many myocardial depressant factors have been identified, including cytokines, components of the complement cascade, pathogen‐associated molecular patterns, endogenous damage‐associated molecular patterns, oxidative stress, altered nitric oxide metabolism, mitochondrial dysfunction, abnormal calcium movement within myocyte apoptosis, and autonomic dysregulation [7, 8]. There are several characteristic pathological aspects of septic cardiomyopathy in this case, warranting discussion:

TKIs, particularly ponatinib, have been linked to endothelial dysfunction and vascular events, including microvascular injury and arterial occlusion [9–11]. This case involves a patient′s TKI; areas with inflammatory cell infiltration exhibit indistinct CD31 and CD34 markers for endothelial cells, suggesting endothelial cell impairment. The result of vascular endothelial cell damage may have led to the infiltration of inflammatory cells. Previous studies have shown that the endothelial glycocalyx is shed in sepsis [12], leading to vascular leak, coagulation, and inflammation, and is associated with adverse outcomes [13]. Once disrupted, the endothelium can result in heterogeneous microvascular flow and myocardial edema [14], representing an underexplored mechanism of SIC. Taken together, the endothelial attenuation observed here is compatible with sepsis‐related myocardial injury; however, given the ongoing TKI exposure and reports of TKI‐associated endothelial dysfunction, a contributory TKI interaction cannot be excluded and should be considered possible.

Secondly, more substantial inflammatory cell infiltration in the region of the old myocardial infarction suggests preexisting vascular endothelial cell damage due to the old myocardial infarction. It is speculated that there was already vascular endothelial cell damage in the area, contributing to a higher incidence of septic cardiomyopathy in that region. This implication aligns with the previous reports [15, 16] indicating an increased risk of septic cardiomyopathy in cases with a history of heart failure, suggesting that the presence of impaired myocardium may contribute to the onset of septic cardiomyopathy.

To elucidate the pathophysiology of septic cardiomyopathy onset and identify contributing factors to inflammatory cell infiltration in this particular case, it is essential to evaluate pathological findings of septic cardiomyopathy occurrence in cases with a background of old myocardial infarction and those involving the use of TKIs. This evaluation should encompass endothelial cell damage, among other aspects. Future reports are anticipated to investigate this matter further.

This case represents SIC in a patient with a prior myocardial infarction receiving ongoing TKI therapy. While septic cardiomyopathy is the primary diagnosis, a contributory effect of TKI exposure cannot be excluded, and the preexisting infarct substrate may have amplified vulnerability. Accordingly, both TKI‐related endothelial dysfunction and the old infarct should be considered potential modifiers of disease expression. Further studies are needed to understand the pathogenesis of SIC.

## Ethics Statement

Research Ethics Committee approval is not required for case reports at our institution.

## Consent

After obtaining informed consent, the postmortem examination was conducted according to the institutional guidelines. Hiroshi Abe obtained the patient consent statement detailing the authorization for the postmortem examination. Consent for publication of the findings was also obtained following the same procedures.

## Disclosure

All authors agree to be accountable for all aspects of the work. Kiyoshi Takasu, Tomita Shigeki, Kei Yamamoto, Tetsuro Miyazaki, Takashi Tokano, and Tohru Minamino gave final approval of the version to be published.

## Conflicts of Interest

The authors declare no conflicts of interest.

## Author Contributions

Hiroshi Abe: substantial contributions to the conception and design of the work and drafting the article. Kiyoshi Takasu, Tomita Shigeki, Kei Yamamoto, and Tetsuro Miyazaki: substantial contributions to the analysis and interpretation of data for the work and critical revision of the article for important intellectual content. Takashi Tokano and Tohru Minamino: substantial contributions to the critical revision of the article for important intellectual content.

## Funding

No funding was received for this manuscript.

## Data Availability

The data that support the findings of this study are available on request from the corresponding author. The data are not publicly available due to privacy or ethical restrictions.
